# Xenon treatment after severe traumatic brain injury improves locomotor outcome, reduces acute neuronal loss and enhances early beneficial neuroinflammation: a randomized, blinded, controlled animal study

**DOI:** 10.1186/s13054-020-03373-9

**Published:** 2020-11-27

**Authors:** Rita Campos-Pires, Haldis Onggradito, Eszter Ujvari, Shughoofa Karimi, Flavia Valeo, Jitka Aldhoun, Christopher J. Edge, Nicholas P. Franks, Robert Dickinson

**Affiliations:** 1 Anaesthetics, Pain Medicine and Intensive Care Section, Department of Surgery and Cancer, Imperial College London, Sir Ernst Chain Building, South Kensington, London, SW7 2AZ UK; 2 Royal British Legion Centre for Blast Injury Studies, Department of Bioengineering, Imperial College London, Bessemer Building, South Kensington, London, SW7 2AZ UK; 3 Charing Cross Hospital Intensive Care Unit, Critical Care Directorate, Imperial College Healthcare NHS Trust, London, UK; 4 Department of Life Sciences, Imperial College London, Sir Ernst Chain Building, South Kensington, London, SW7 2AZ UK; 5 Department of Anaesthetics, Royal Berkshire Hospital NHS Foundation Trust, London Road, Reading, RG1 5AN UK

**Keywords:** Xenon, Noble gases, Neuroprotection, Neurotrauma, Acquired brain injury, Neuroinflammation, Neuroglia, Locomotor deficit

## Abstract

**Background:**

Traumatic brain injury (TBI) is a major cause of morbidity and mortality, but there are no clinically proven treatments that specifically target neuronal loss and secondary injury development following TBI. In this study, we evaluate the effect of xenon treatment on functional outcome, lesion volume, neuronal loss and neuroinflammation after severe TBI in rats.

**Methods:**

Young adult male Sprague Dawley rats were subjected to controlled cortical impact (CCI) brain trauma or sham surgery followed by treatment with either 50% xenon:25% oxygen balance nitrogen, or control gas 75% nitrogen:25% oxygen. Locomotor function was assessed using Catwalk-XT automated gait analysis at baseline and 24 h after injury. Histological outcomes were assessed following perfusion fixation at 15 min or 24 h after injury or sham procedure.

**Results:**

Xenon treatment reduced lesion volume, reduced early locomotor deficits, and attenuated neuronal loss in clinically relevant cortical and subcortical areas. Xenon treatment resulted in significant increases in Iba1-positive microglia and GFAP-positive reactive astrocytes that was associated with neuronal preservation.

**Conclusions:**

Our findings demonstrate that xenon improves functional outcome and reduces neuronal loss after brain trauma in rats. Neuronal preservation was associated with a xenon-induced enhancement of microglial cell numbers and astrocyte activation, consistent with a role for early beneficial neuroinflammation in xenon’s neuroprotective effect. These findings suggest that xenon may be a first-line clinical treatment for brain trauma.
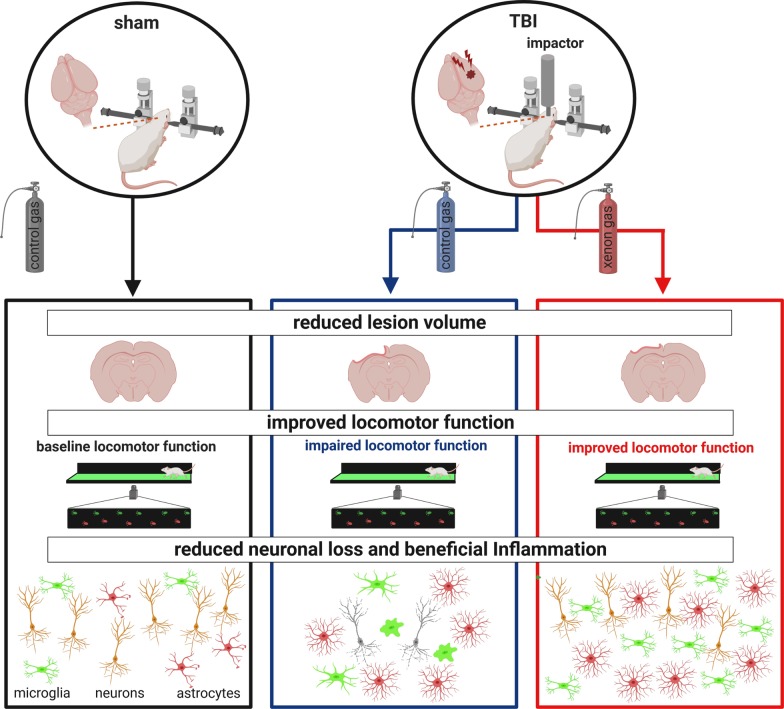

## Introduction

Traumatic brain injury (TBI) is a leading cause of death and disability globally [1, 2]. It is estimated that annually there are up to 60 million TBIs worldwide [3]. TBI results from an external mechanical force causing *primary injury* that initiates a complex biochemical and cellular pathophysiology leading to *secondary injury* developing in the minutes, hours, and even months later. Many of the persistent impairments and disabilities experienced by TBI survivors are caused by the potentially preventable secondary injury. Despite a greater understanding of the pathophysiology of TBI in recent years, current treatment is largely supportive, with no clinically proven treatments specifically targeting neuronal loss and secondary injury development*.*

Xenon is a noble gas used medically as a general anesthetic and in MRI imaging [4, 5]. Xenon is a pleiotropic drug with actions at a variety of targets implicated in the secondary injury cascade, including NMDA receptors [6–8], potassium channels [9, 10], activation of HIF-1 alpha [11], and an increase in erythropoietin levels [12]. Xenon has been shown to be neuroprotective using in vitro and in vivo models of ischemic brain injury [4, 13–19], and a recent two-center clinical trial of xenon for brain injury after out-of-hospital cardiac arrest showed evidence of reduced cerebral white matter damage [20]. Until recently, the efficacy of xenon as a neuroprotectant in TBI has been limited to simple in vitro models [21–24]. We recently demonstrated for the first time in an animal model that xenon is neuroprotective following moderate TBI in mice [25]. Clinically TBI is highly heterogeneous and from a translational perspective it is important to evaluate neuroprotection in different injury severities and other species [26]. Xenon has not previously been evaluated in rats after TBI. In the current study, the objective was to evaluate the effect of xenon treatment following severe TBI in rats, with a focus on acute functional outcome, neuronal preservation, and glial cell responses in specific brain regions associated with the cognitive, locomotor and other functional deficits experienced by TBI patients.

## Materials and methods

Experiments complied with the UK Animals Scientific Procedures Act (1986) and were approved by the Animal Welfare and Ethical Review Body of Imperial College London. Unless otherwise stated, reagents were purchased from Sigma Aldrich (Dorset, UK).

We designed our study to comply with the ARRIVE guidelines [27]. Young adult male Sprague Dawley rats *n* = 22, age 13 weeks, mean weight (SEM) 429 (7) g at the time of surgery were obtained from Charles River (Margate, Kent, UK). All animals had undergone no previous procedures before entering this study. Animals were group housed (4 per cage) in filter-top cages in a pathogen-free facility in a 12:12 light/dark cycle (7am–7pm light) at 22 °C with ad libitum access to food and water. Animals were monitored daily before experiments, and closely monitored in the postoperative period for at least 4 h, and then early the following day.

### Experimental groups, randomizing and blinding

Animals were randomly assigned to TBI primary injury (no treatment) or TBI followed by 50% xenon:25% oxygen balance nitrogen or TBI followed by 75% nitrogen:25% oxygen (control gas) or sham surgery followed by 75% nitrogen:25% oxygen (control gas) groups. The experimenter performing the surgery was blinded to treatment. A separate experimenter, blinded to groups and treatment, performed behavioural tests. All histological outcomes were assessed by blinded observers. Animals were allowed to survive for 15 min (primary injury group) or 24 h after injury. We had six animals in each 24-h group (TBI control; TBI xenon; sham surgery) and four animals in the primary injury group. Sample sizes were based on power calculations using effect sizes observed previously after moderate TBI in mice [25]. The lesion volume of the primary injury group was used to calculate the secondary lesion volume at 24 h.

### Traumatic brain injury

Animals were anesthetized with 2.5% isoflurane with buprenorphine analgesia (0.04 mg kg^−1^) in an air/oxygen mixture (35% oxygen:65% nitrogen) supplied via a facemask in spontaneously breathing animals. Core body temperature was monitored and maintained at 37 °C for the duration of the surgery by means of a rectal probe and feedback-controlled heating pad (CMA450, CMA Microdialysis AB, Solna, Sweden). Temperature, pulse oximetry and heart rate were measured throughout. Traumatic brain injury was performed using a Leica ImpactOne (Leica Biosystems, Milton Keynes, UK) controlled cortical impact device. Animals were fixed in a stereotactic frame and after being given subcutaneous lidocaine (2 mg kg^−1^), a scalp incision was made followed by a craniotomy. A craniotomy window (~ 8 mm × 6 mm) was created using a saline-cooled high-speed drill, along the coronal and lambdoid sutures and laterally as close as possible to the temporalis muscle insertion. The bone flap was removed exposing the dura above the right parietal cortex, between the sagittal, lambdoid, and coronal sutures. The tip of the controlled cortical impact device was positioned anteriorly above the intact dura ~ 1 mm from sagittal suture. The angle of the impactor, approximately 25 degrees from sagittal plane, was adjusted such that the tip was perpendicular to the dural surface. The impactor tip was flat, with a diameter of 4 mm, impact velocity of 6 m s^−1^, impact duration of 400 ms, and penetration depth of 3.0 mm. Our CCI impact parameters and the functional and histological outcomes are similar to those classified as a severe injury [28]. Following CCI surgery, the craniotomy was closed with the bone flap, sealed with tissue glue (Histoacryl, Braun-Melsungen, Melsungen, Germany) and dental cement (Poly-F Plus, Dentsply Sirona, UK) and the skin sutured. Sham-surgery animals underwent identical anesthesia, temperature control, placement in stereotactic frame, surgical skin incision to reveal the surface of the skull which was drilled superficially but no craniotomy was performed. The duration of the sham surgery and anesthesia was identical to that of the CCI animals. The choice of anesthetic and analgesic drugs in animal TBI studies may have an impact on how secondary injury develops [29–31]. In order to avoid any confounding effects from the anesthesia and analgesia, we were careful to ensure that the sham group received exactly the same drugs. For our study, we chose to combine the widely used inhalational anesthetic isoflurane and the long-acting opioid buprenorphine both widely used, safe and effective in rodents [32, 33].

### Xenon or control gas administration

Gas treatments were administered to spontaneously breathing animals in a series of custom-made chambers linked in a closed circuit for a total duration of three hours, starting 30 min after CCI injury. One animal in the xenon group had the treatment start time delayed to 1 h in error. Data from this animal are not included in the study and the animal was replaced. Gas concentrations inside the circuit were monitored continuously via a xenon meter (model 439 EX, Nyquist Ltd, UK) and an oxygen meter (Oxydig, Draeger, Luebeck, Germany) included in the circuit. Carbon dioxide was removed from the system by soda lime pellets. Additional volumes of gases were added as necessary to maintain their respective concentrations within the range 21–25% for oxygen and 45–50% for xenon throughout the 3-h administration period. Gases were circulated using a small animal ventilator (SAR-1000 Small Animal Ventilator, CWE Incorporated, Ardmore, United States). Xenon (BOC HiQ 49.96% xenon:25.03% oxygen:25.01% nitrogen) and control gas (25% oxygen:75% nitrogen) were obtained from BOC Ltd, Guildford, UK. The animals’ temperature was monitored using a rectal temperature probe before and after administration of gases and was within normal physiological range. Following the 3-h treatment period with xenon or control gas, animals were returned to a home cage where they breathed room air.

### Functional outcomes

The CatWalk-XT automated gait analysis system (Noldus Information Technology, Wageningen, the Netherlands) was used to measure locomotor function and gait parameters. Animals underwent baseline testing before injury or sham procedure, and again 24 h following TBI or sham surgery. The system consists of a runway with glass plate floor with dim light illuminating the glass from the side. In a darkened environment (< 1 lx of illumination), light is reflected downward when the animal’s paws contact the glass surface. Animals walk spontaneously along the runway toward a goal box. Images of the footprints are recorded by a video camera under the walkway. Three consecutive trials were performed for each animal. The images from each trial were processed and analyzed on a computer by Catwalk-XT software and the mean value of the gait parameters obtained.

### Histological processing

At 15 min or 24 h, animals were terminally anesthetized with pentobarbital and transcardially perfused with 50 ml of cold PBS followed by 300 ml of cold 4% paraformaldehyde (ThermoFisher Ltd, Hemel Hempstead, Herts, UK). Brains were carefully removed from the skull and left in 4% paraformaldehyde (in PBS) overnight at 4 °C, then transferred to 30% sucrose in PBS until the brains sank, before being frozen on powdered dry ice. Frozen brains were embedded in Optimal Cutting Temperature mounting medium (Cell Path Ltd, Newton, Powys, UK) and cut in the coronal plane with a cryostat tissue slicer (Leica CM3050). To quantify lesion volume, for each brain, a total of 30–34 sections (20 µm thick) spanning the entire lesion were collected on Superfrost® Plus microscope slides (ThermoFisher Ltd, Hemel Hempstead, Herts, UK) every 500 µm.

### Quantification of contusion volume

Slices (20 µm thick) were stained with cresyl violet (Acros Organics, Fisher Scientific, UK), as described previously [25]. Slices were imaged with a digital camera (Scopetek DCM510, Scopetek Opto-Electric Co., Hangzhou, China) attached to a stereomicroscope (Wild model M8, Heerbrugg, Switzerland). The contusion was evident from a clear difference in the intensity of the cresyl-violet staining. The area of the contusion was measured using image-analysis software (Scopephoto 3.1, Scopetek Opto-Eletric Co., Hangzhou, China) by an investigator blinded to the experimental groups. Contusion volume was calculated by multiplying contusion areas, A, by the distance between brain sections, d, (500 µm), according to the following formula:

$$\frac{d}{2}*\left({A}_{1 \, }+{A}_{n}\right)+d*\left({A}_{2 \, }+{A}_{3}+\dots +{A}_{n-1}\right)$$

Secondary injury volume at 24 h was calculated by subtracting the mean primary injury contusion volume at 15 min from the total contusion volume measured at 24 h.

*Immunofluorescence staining.* Twenty-micrometer-thick slices from the perfused brains were used for immunofluorescence staining for NeuN (neurons), Iba1 (microglia), GFAP (reactive astrocytes), and DAPI (nuclei). The antibodies and dilutions used were: NeuN (1:200 mouse, clone A60 AlexFuor555 conjugate, MAB 377A5, Merck-Millipore, Watford, Herts, UK); Iba1 (primary: 1:200 rabbit anti-rat, C292720 Lifespan Biosciences, Inc, Seattle, USA; secondary: 1:500 AlexaFluor488 goat anti-rabbit, A11008, Life Technologies, Paisley, UK); GFAP (primary: 1:1000 chicken, AB4674, Abcam Ltd, Cambridge, UK; secondary: 1:200 goat anti-chicken, AlexaFluor647, AB150175, Abcam Ltd, Cambridge, UK). Slices were washed in PBS + 0.3% TritonX100) and blocked for 1.5 h with 10% normal goat serum (diluted in PBS-0.3% Triton) at room temperature. Sections were incubated overnight at 4 °C with the conjugated and primary antibodies in blocking solution. The following day, sections were washed with PBS-0.3% Triton three times, for 20 min each, and incubated for 1 h at room temperature with the secondary antibodies. Sections were washed with PBS-0.3% Triton three times, for 20 min each, and mounted with Vectashield with DAPI (H1200, Vector Laboratories, Peterborough, UK) and glass coverslips (Menzel-Gläser, 22 × 60 mm №1 BB022050A1).

### Imaging and analysis

Images were captured with a Zeiss AxioObserver inverted widefield microscope (Facility for Imaging by Light Microscopy, Imperial College London) equipped with a motorized stage and a 20 × objective (Zeiss Plan Apochromat, NA 0.8, WD 0.55 mm). The whole slice area was imaged using the multi-position acquisition function of Zeiss Zen software (LED excitation wavelengths 365 nm, 470 nm, 555 nm, and 625 nm). The acquisition focal plane corresponded to the image maximal sharpness (best focus) at five different areas of the brain slice. Images were analyzed with FIJI (ImageJ) software [34, 35]. The four channels were separated and scaled. NeuN-, Iba1-, and GFAP-positive staining was quantified in the contralateral primary motor/association cortex (M1/MPtA), and bilaterally in the retrosplenial cortex (RSC), barrel field of somatosensory cortex (S1BF), amygdala, ventromedial hypothalamus and hippocampal CA1, CA2, CA3 and DG subregions by observers blinded to the experimental groups. Due to tissue damage within the contusion and disruption of cortical layers, it was not possible to quantify neurons in the ipsilateral motor/association cortex. For the neuronal counting in the cortical regions, we used rectangular regions of interest of width 200 µm spanning cortical layers 1 to 6, a circle of diameter 600 µm in the amygdala, an oval (520 × 670 µm) in the hypothalamus; in the hippocampus we used the following rectangular regions of interest: CA1 (300 × 30 µm), CA2 (200 × 70 µm), CA3 (250 × 50 µm), DG (two 200 × 50 µm on top; one 200 × 55 µm on bottom). Neurons were manually counted using FIJI (ImageJ), in two slices per brain and the mean density calculated. For the quantification of microglia and astrocytes we, used circular regions of interest in the left M1/MPtA (1300 µm diameter), S1BF (1300 µm diameter), amygdala (600 µm diameter), the contusional cortex (700 µm diameter); oval regions in the RSC (1300 × 600 µm) and ventromedial hypothalamus (561 × 636 µm). Due to tissue damage in the contusion it was not always possible to position the contusional cortex ROI in exactly the same anatomical area; the ROI was always within the contusion, in either: M1/MPtA (6/6 sham; 1/4 TBI control; 2/5 TBI xenon), edge of S1BF (1/5 TBI xenon) or edge of RSC (3/4 TBI control; 2/5 TBI xenon); in all cases there was no overlap with the ROIs for S1BF or RSC; in 2 of the TBI control group it was not possible to find a non-overlapping ROI within the contusion and these were excluded. In the hippocampus, outlines of total CA1, CA2, CA3 and DG regions were drawn for each slice using the ImageJ line tool; in the corpus callosum, the outline of the central area was drawn for each slice. To quantify the number of Iba1-positive cells; the background was subtracted (Gaussian blur function), the image was binarized and particles with an area of 40 µm^2^ or larger were counted using automated or manual counting. We classified microglia based on their morphology; resting microglia have smaller rounder soma with high ramification, while activated microglia assume hypertrophic or bushy phenotypes with a larger more amorphous soma with less ramification [36, 37]. We used a quantitative method using the roundness and size of the cell soma in order to classify all the microglia in each ROI as resting or active, with smaller round cells (area < 100 µm^2^; roundness > 0.5) classified as low activity or resting, and larger irregular cells (area > 100 µm^2^; roundness < 0.5) classified as active microglia [38]. To quantify reactive astrogliosis, we measured the area of GFAP positive staining within the regions of interest; the GFAP images were binarized after thresholding and the percentage of GFAP stained area within the regions of interest was measured. In all of the immunohistological measurements, TBI control and TBI xenon groups were compared with the sham group that had been treated identically to the TBI groups but without impact, in order to ensure that any effects are independent of the drugs administered or surgery. One of the immunohistology slides from the xenon group was damaged and could not be imaged. Due to tissue damage or imperfections such as folds, it was not possible to make neuronal count measurements in every ROI (*eg* right RSC, left & right hypothalamus) from every animal (individual points are shown on the graphs).

### Statistics

Data were assessed for normality using the Shapiro–Wilk test. We assessed significance of differences in contusion volume using a Mann–Whitney U test. The locomotor function data was normally distributed and was analyzed using ANOVA test with Sidak correction. Normality tests, ANOVA and Mann–Whitney tests were implemented using GraphPad Prism Version 7.03 software (GraphPad Software Inc., La Jolla, CA). Some of the NeuN-, Iba1- and GFAP-positive distributions in the ROIs were found to be significantly different from a normal distribution and could not be transformed into a normal distribution. Therefore, for all the immunohistology the regions of interest in the TBI control, TBI xenon and sham groups were compared using a Kruskal–Wallis (KW) test with Benjamini Yekutieli correction implemented using the statistical program Stata (Version 15, StataCorp, College Station, Texas). As the null statistics for the KW test are known not to follow a chi-squared distribution for small numbers especially in the region of the 0.95 and 0.99 quantile, results from the KW test were compared to the exact results for a KW test using a program written in Mathematica (Mathematica 11.3.0.0, Wolfram Research Inc.) [39]. P values of 0.05 or less were taken to indicate a significant difference. Values are quoted as mean (SEM) for normally distributed data or median (IQR) if data are not normally distributed. The experimental unit *(n)* in all outcomes represents an animal. The sample sizes (*n*) are indicated in the figure legends.

## Results

### Xenon reduces secondary injury volume at 24 h

**Fig. 1:**
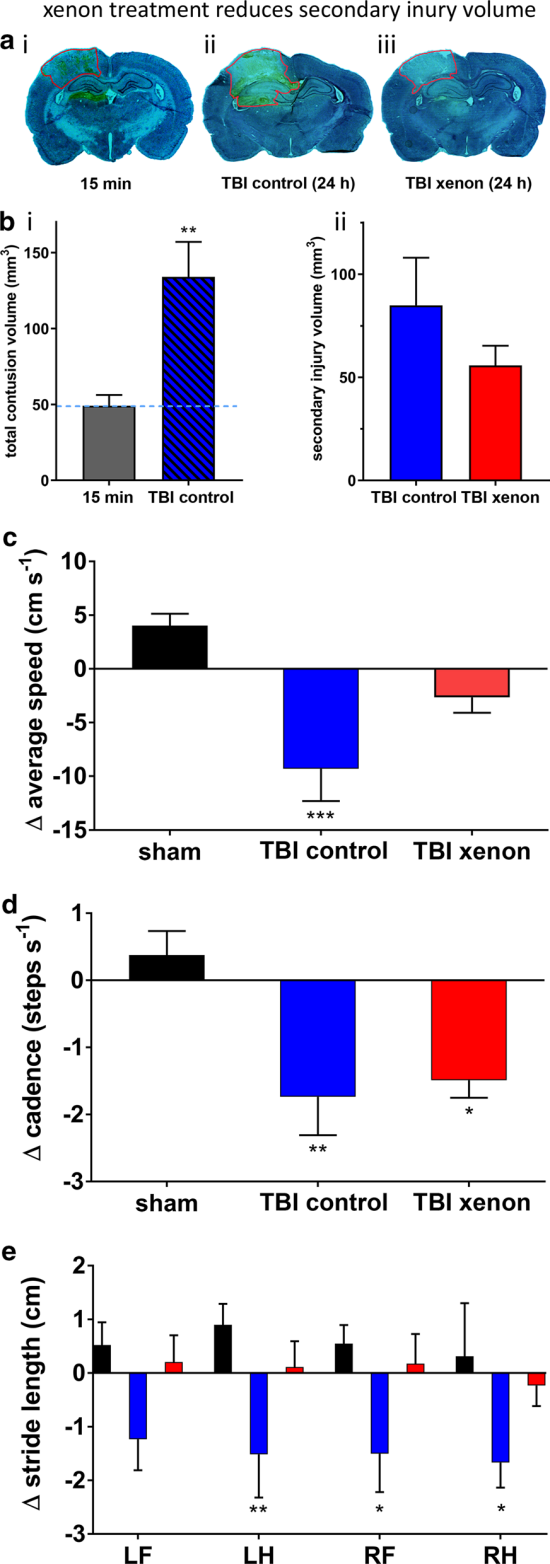
Xenon treatment reduces secondary injury development. Controlled cortical impact results in a primary injury that develops significantly 24 h later. **a** Typical cresyl violet stained slices for **i** TBI primary injury at 15 min, **ii** TBI control at 24 h and **iii** TBI xenon at 24 h. **b(i)** In animals treated with control gas, the injury develops significantly between 15 min (grey bar) and 24 h (dark blue hatched bar). The area above the dashed-line represents the secondary injury. **ii** Treatment with xenon (50%) (red bar) resulted in a 34% reduction in secondary injury compared to untreated control (dark blue bar). Secondary injury was calculated by subtracting the primary injury at 15 min from the total contusion volume at 24 h. **c** Controlled cortical impact results in locomotor impairment at 24 h after injury that is prevented by xenon treatment. There was a significant reduction in locomotor speed in the TBI control group at 24 h, while this reduction was absent in the TBI xenon group. **d** Cadence was significantly reduced in both the TBI control group and the TBI xenon group at 24 h following injury. **e** Stride length is reduced following TBI in the TBI control group but not in the TBI xenon group. LF left front paw; LH left hind paw; RF right front paw; RH right hind paw. Bars are mean values, error bars are SEM. * p < 0.05, ** p < 0.01, *** p < 0.001 compared to sham group, Mann Whitney U test (contusion), one-way (locomotor speed, cadence) or two way (stride length) ANOVA with Sidak correction. n = 4, primary injury 15 min (grey bar); n = 6 sham (black bars) 24 h, n = 6, TBI control 24 h (blue bars); n = 6 TBI xenon 24 h (red bars)

Our controlled cortical injury parameters resulted in a primary lesion at 15 min of volume 49 (7) mm^3^, mean (SEM), that developed significantly (p < 0.01) increasing to 134 (23) mm at 24 h after injury (Fig. 1a(i),(ii), b(i), representing a 2.7-fold increase. Secondary injury volume at 24 h, calculated by subtracting the primary lesion volume at 15 min, was decreased by 34% in the xenon-treated group, although this did not reach statistical significance (Fig. 1b(ii)). Given that the lesion volume in the severe injury in rats appears to have a greater variance than the moderate injury that we previously investigated in mice, it is possible that with larger group sizes a statistically significant difference might have been observed.

### Xenon reduces acute locomotor impairment

We assessed locomotor function at baseline in sham, TBI control and TBI xenon groups before CCI or sham surgery, and again 24 h after injury or sham procedure. In order to increase sensitivity and to observe differences in individual animal performance we calculated the change in each parameter (Δ) at 24 h compared to the same animal at baseline. At 24 h the sham group exhibited a small increase in locomotor speed of 4.0 (1.1) m s^−1^ compared to baseline, perhaps indicating a learning effect (Fig. 1c). In contrast in the TBI control group there was a significant (p < 0.05) reduction in locomotor speed, by 9.3 (3.0) m s^−1^, 24 h after injury (Fig. 1c). Interestingly the locomotor speed following injury was not significantly different in the xenon-treated TBI group (Fig. 1c). Cadence was significantly (p < 0.05) reduced in both the TBI control and TBI xenon groups (Fig. 1d). In order to investigate locomotor impairment further and determine whether there was any lateralization we examined the stride-length in individual limbs. Stride length at 24 h was reduced in all limbs in the TBI control group, reaching significance (p < 0.05) in the left hind, right front, and right hind limbs (Fig. 1e). In contrast, stride length was not significantly changed in the TBI xenon group (Fig. 1e).

### Xenon reduces neuronal cell loss in key brain regions

**Fig. 2:**
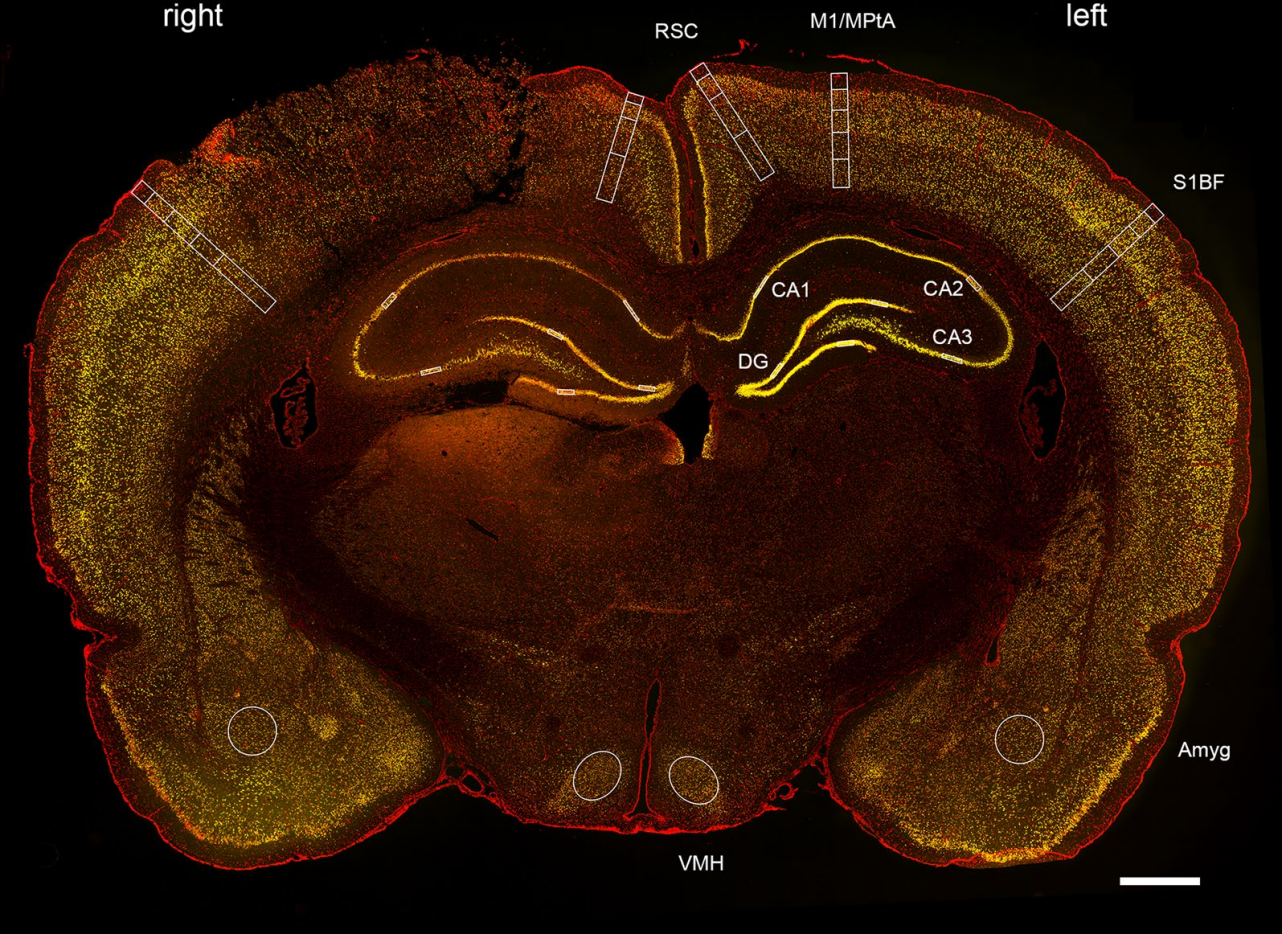
Neuronal loss was quantified in coronal brain sections. Image shows a typical section at Bregma -3.12 mm from a xenon-treated TBI animal at 24 h, stained with the neuronal marker NeuN (yellow) and nonspecific nuclear marker DAPI (red). The contusion is visible in the right hemisphere motor area (left of image). Neurons were counted in the left and right retrosplenial cortex (RSC) in layers 1, 234, 5 & 6; the left motor/medial parietal association cortex (M1/MPtA) in layers 1, 23, 4, 5 & 6; left and right somatosensory cortex (S1BF) in layers 1, 23, 4, 5 & 6; and in the right and left subcortical regions of hippocampus (CA1; CA2; CA3 & DG), amygdala (Amyg) and ventromedial hypothalamus (VMH). The scale bar is 1000 μm

We assessed whether our controlled cortical impact injury resulted in neuronal loss in clinically relevant brain regions and whether xenon treatment could prevent or attenuate this loss. We chose to examine cortical and subcortical brain regions (Fig. 2) chosen to include both pericontusional areas and areas distant from the lesion core and that are associated with functional impairment observed following TBI. We observed neuronal loss in the TBI control group compared to uninjured sham group that was not present in the TBI xenon group.

### Cortical neuronal loss

**Fig. 3:**
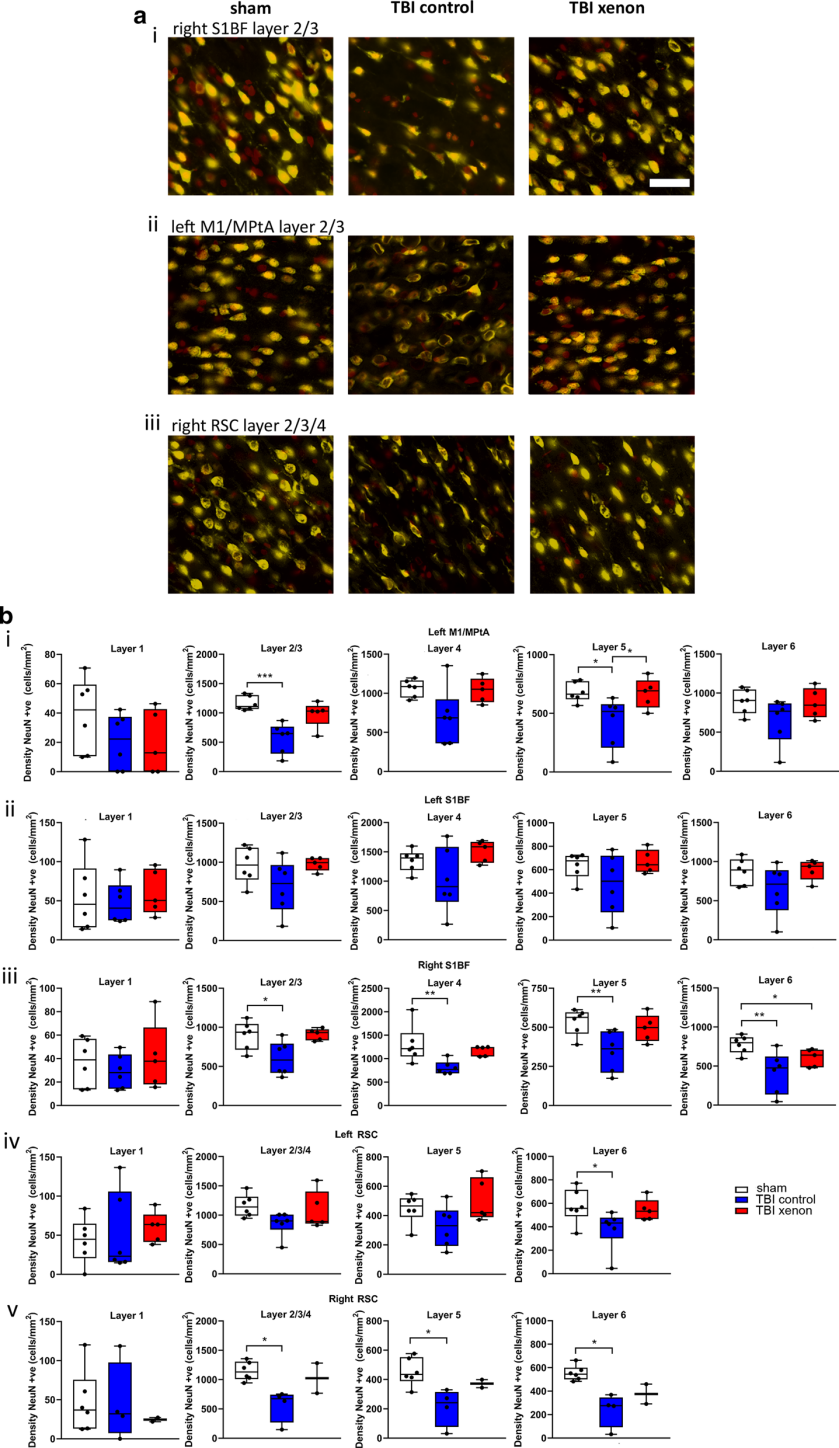
**a** Typical immunostaining showing NeuN (yellow) and DAPI (red) staining from sham, TBI control and TBI xenon animals in **i** right somatosensory cortex layers 2/3, **ii** left motor/association cortex layers 2/3 and **iii** right retrosplenial cortex layers 2/3/4. Live neurons show a strong NeuN staining combined with DAPI. The scale bar is 20 μm and applies to all images. **b** Xenon prevents neuronal loss in specific cortical regions 24 h after TBI. Quantification of neuronal cell density of cortical layers from sham (white bars), TBI control (blue bars) and TBI xenon (red bars) in **i** left motor/medial parietal association cortex (M1/MPtA), **ii** left somatosensory cortex (S1BF), **iii** right somatosensory cortex (S1BF), **iv** left retrosplenial cortex (RSC), **v** right retrosplenial cortex (RSC). The lines are medians, boxes represent interquartile interval and whiskers are range. * p < 0.05; ** p < 0.01; *** p < 0.001, compared to sham group or control TBI group as indicated by brackets, Kruskal Wallis test with Benjamini Yekutieli correction. n = 6 sham (white boxes) 24 h, n = 6, TBI control 24 h (blue boxes); n = 5 TBI xenon 24 h (red boxes)

Figure 3a shows representative images of NeuN stained neurons in (i) layers 2 & 3 of the right somatosensory cortex and (ii) the left motor cortex and (iii) layers 2, 3 & 4 of the right retrosplenial cortex from sham, TBI control and TBI xenon groups exhibiting neuronal loss in TBI control group that is absent in TBI xenon group. Quantification of neurons (Fig. 3b) in the left motor cortex showed significant (p < 0.05) loss in the TBI control group compared to the sham group in the TBI control group in layers 2 & 3, and layer 5, that was prevented in layer 5 and reduced in layers 2 &3 in the xenon-treated TBI group (Fig. 3b(i)). In layer 4 and layer 6 of the left motor cortex a reduction in median neuronal density in the TBI control group was evident compared to sham group but this did not reach significance (Fig. 3b(i)). The median cell density in the xenon-treated groups in these layers was not different to sham. In the left somatosensory cortex, the median value of neuronal density in the TBI control group was less than the sham group but this did not reach significance, while the median value of the xenon-treated group was similar to that of the uninjured sham group ((Fig. 3b(ii)). In the pericontusional right somatosensory cortex, there was significant (p < 0.05) neuronal loss in the TBI control group in layers 2 & 3, layer 4, layer 5 and layer 6 (Fig. 3b(iii)). Xenon treatment reduced neuronal loss in layers 2 & 3, layer 4, layer 5 and layer 6. In the left retrosplenial cortex there was a significant (p < 0.05) loss of neurons in layer 6 of the TBI control group that was absent in the xenon-treated TBI group ((Fig. 3b(iv)). In layer 2, 3 & 4 and layer 5 the median neuronal density in the TBI control group was reduced compared to sham but did not reach significance, while the median neuronal density in the TBI xenon group was similar to that of the sham group. In the pericontusional right retrosplenial cortex there was a significant (p < 0.05) loss of neurons in the TBI control group in layers 2,3,4, layer 5 and layer 6 ((Fig. 3b(v)).

### Subcortical neuronal loss

**Fig. 4:**
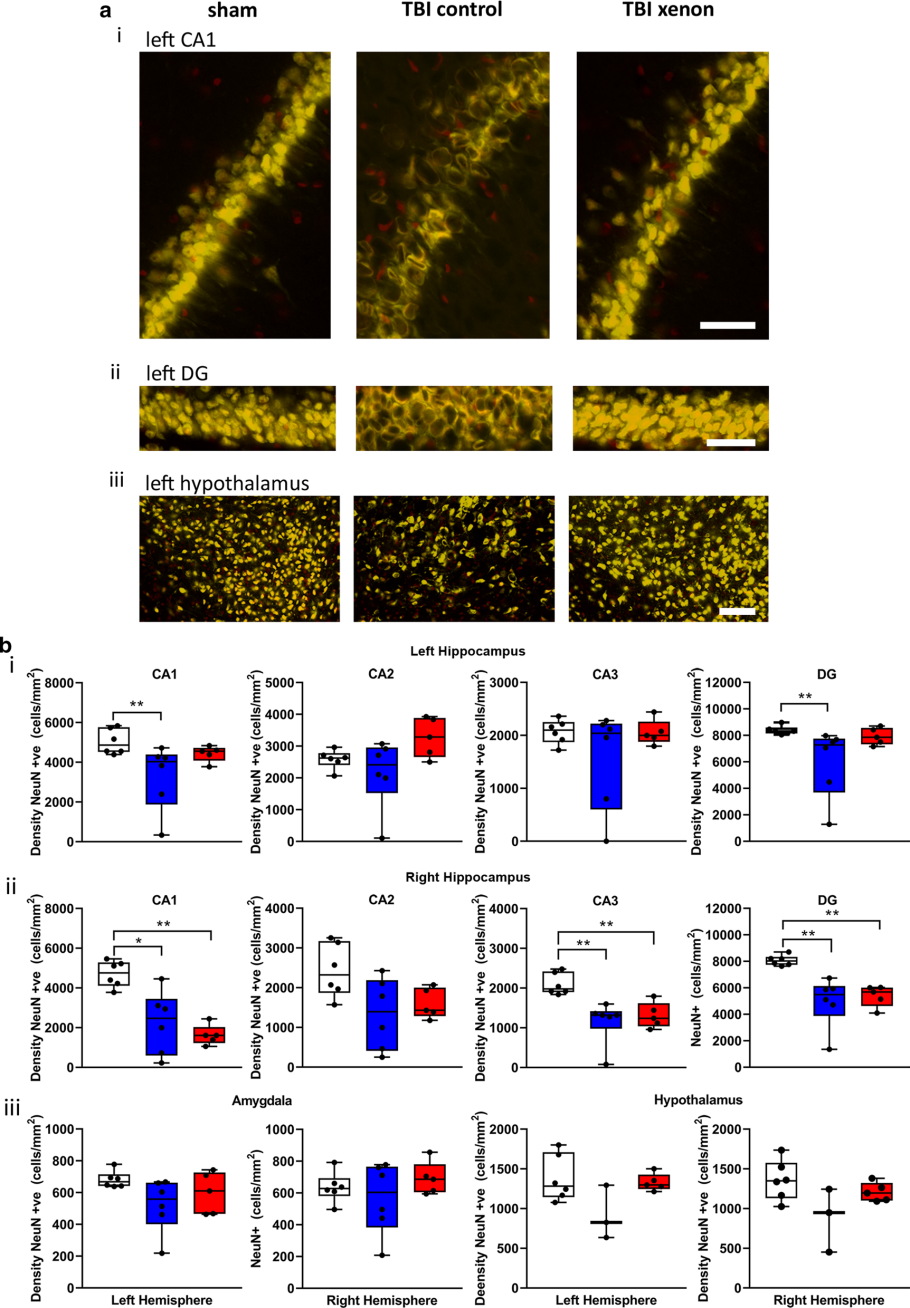
**a** Typical immunostaining showing NeuN (yellow) and DAPI (red) staining from sham, TBI control and TBI xenon animals in **i** left CA1 hippocampal region, **ii** left dentate gyrus **iii** left hypothalamus. Live neurons show a strong NeuN staining combined with DAPI. The scale bars are 20 μm (i) and (ii); and 40 μm (iii). **b** Xenon prevents neuronal loss in specific subcortical regions 24 h after TBI. Quantification of neuronal cell density of cortical layers from sham (white bars), TBI control (blue bars) and TBI xenon (red bars) in **i** left hippocampal CA1, CA2, CA3 and DG regions, **ii** right hippocampal CA1, CA2, CA3 and DG regions, **iii** left and right amygdala, left and right hypothalamus. The lines are medians, boxes represent interquartile interval and whiskers are range. * p < 0.05, ** p < 0.01, compared to sham group as indicated by brackets, Kruskal Wallis test with Benjamini Yekutieli correction. n = 6 sham (white boxes) 24 h, n = 6, TBI control 24 h (blue boxes); n = 5 TBI xenon 24 h (red boxes)

Figure 4a shows representative images of NeuN stained neurons in the (i) left hippocampal CA1 region, (ii) left hippocampal dentate gyrus region and (iii) left hypothalamus from sham, TBI control and TBI xenon groups showing neuronal loss in TBI control group that is absent in TBI xenon group. Quantification of neuronal loss in the left hippocampus showed significant (p < 0.05) neuronal loss in the TBI control group in the CA1 and DG sub-regions that was reduced by xenon treatment (Fig. 4b(i)). In the left CA2 and CA3 sub-regions the median neuronal density of the TBI control group was lower than the sham group but this did not reach significance (Fig. 4b(i)). In contrast, in the right hippocampus there was a significant (p < 0.05) neuronal loss in both the TBI control group and the TBI xenon group in the CA1, CA3 and DG sub-regions (Fig. 4b(ii)). In the left and right amygdala and hypothalamus, there was a reduction in median neuronal density in the TBI control group compared to the sham group but this did not reach significance, while the median neuronal density in the TBI xenon group was similar to the sham value (Fig. 4b(iii)).

### Xenon treatment enhances early microglial proliferation in functionally relevant cortical regions

**Fig. 5:**
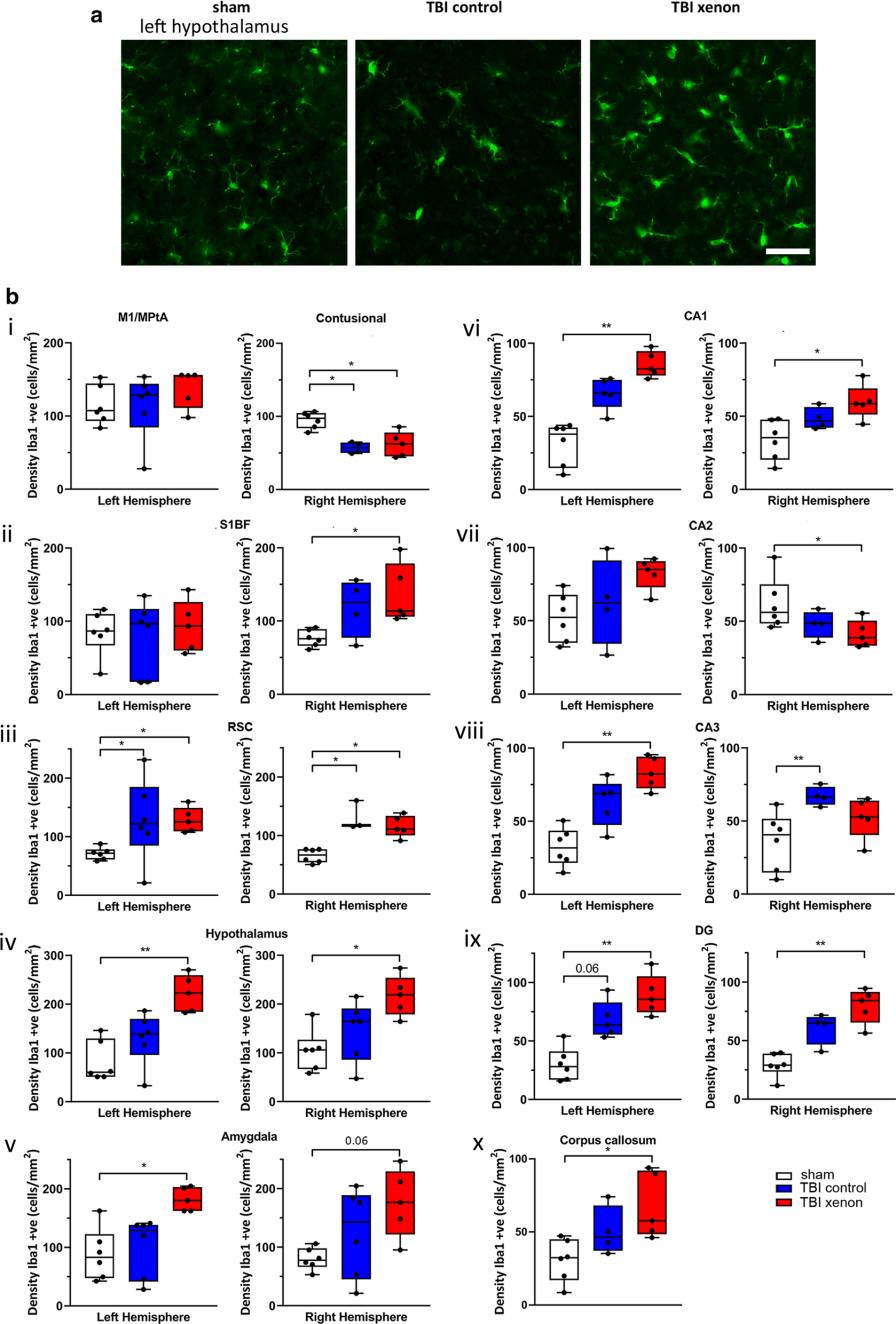
Xenon treatment enhances early microglial proliferation. **a** Typical immunostaining showing Iba1 (green) staining from sham, TBI control and TBI xenon animals in left hypothalamus. The scale bar is 50 μm and applies to all images. **b** Quantification of Iba1-positive cells from sham (white bars), TBI control (blue bars) and TBI xenon (red bars) in **i** motor/medial parietal association cortex (M1/MPtA) & contusional cortex, **ii** somatosensory cortex (S1BF), **iii** retrosplenial cortex (RSC) **iv** hypothalamus, **v** amygdala, **vi** hippocampal CA1, **vii** CA2, **viii** CA3, **ix** dentate gyrus (DG) and **x** corpus callosum. * p < 0.05, ** p < 0.01, compared to sham group as indicated by brackets, Kruskal Wallis test with Benjamini Yekutieli correction. n = 6 sham (white boxes) 24 h, n = 6, TBI control 24 h (blue boxes); n = 5 TBI xenon 24 h (red boxes)

Figure 5 shows quantification of Iba1-positive microglia cortical & subcortical regions. In the cortical areas (Fig. 5b) there was a significant (p < 0.05) increase in median number of Iba1-positive microglia in the xenon-treated group compared to the sham group in the right somatosensory cortex (Fig. 5b(ii)) and bilaterally in the retrosplenial cortex (Fig. 5b(iii)). In the left somatosensory cortex and left motor/association cortex, there was no significant difference between xenon and sham groups. In the left retrosplenial cortex, there was a significant increase in the control TBI group compared to the sham group. In all other areas there was no significant difference between the control TBI group and the sham group or the xenon treated group. In contrast to all other regions, in the contusional cortex, there was a significant (p < 0.05) decrease in median number of microglia in both the TBI control group and the xenon-treated group compared to sham, most likely reflecting the gross tissue loss in this region (Figs. 1, 2).

### Xenon treatment enhances early microglial proliferation in subcortical regions

Figure 5a shows representative Iba1-positive microglia in the left hypothalamus from sham, TBI control and TBI xenon groups. In the left and right CA1 and DG hippocampal subregions, and in the left CA3 subregion there was a significant (p < 0.05) increase in median number of microglia in the xenon-treated group compared to the sham group (Fig. 5b(vi), (viii), (ix)). In all hippocampal subregions except CA3, there was an increase in the median number of microglia in TBI control group compared to the sham group, but this only reached significance (p < 0.01) in the right CA3. In all subcortical regions except the right CA2 and right CA3, the median value in the xenon TBI group was greater than the TBI control group, but this increase did not reach significance. Interestingly in the right CA2 there was a small decrease in the median number of microglia in the TBI control and TBI xenon groups compared to sham, that reached significance (p < 0.05) in the TBI xenon group. Similar findings were observed in the amygdala and hypothalamus with significant (p < 0.05) increases in the xenon-treated group bilaterally in the hypothalamus and in the left amygdala. In the corpus callosum xenon significantly (p < 0.05) increased the median number of Iba1-positive microglia.

### Xenon treatment preferentially enhances the number of low activity or resting microglia in regions where neuronal loss is reduced

**Fig. 6:**
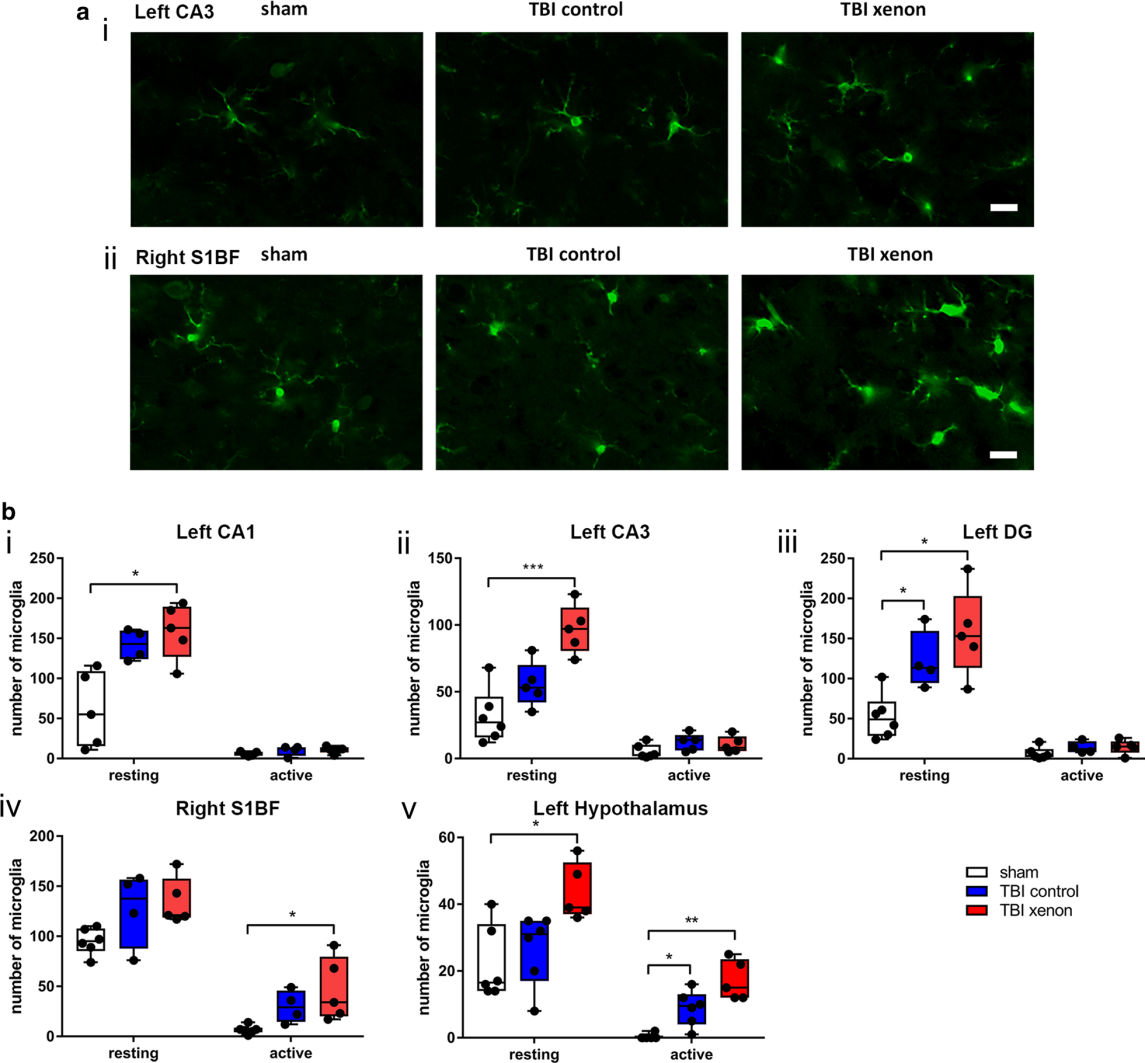
**a** Typical images showing microglial morphology from sham, TBI control and TBI xenon. **i** In the left hippocampal CA3, smaller more round (resting) microglia predominate in all groups with increased numbers in xenon-treated group. **ii** In the right somatosensory cortex (S1BF), smaller more round (resting) microglia predominate in the sham and control TBI groups, while in the xenon TBI group there is an increase in number of larger less ramified and less round (active) microglia. The scale bars are 20 μm and applies to all images. **b** Quantification of resting and active microglia in **i** left hippocampal CA1, **ii** CA3, **iii** DG, **iv** right S1BF and **v** left hypothalamus. The lines are medians, boxes represent interquartile interval and whiskers are range. n = 6 sham (white boxes); n = 6, TBI control (blue boxes); n = 5 TBI xenon (red boxes) * p < 0.05, ** p < 0.01, *** p < 0.001, compared to sham group as indicated by brackets, Kruskal Wallis test with Benjamini Yekutieli correction

Typical examples of microglial morphology in left CA3 and right S1BF are shown in Fig. 6a. In the left CA3 smaller, round (soma area < 100 µm^2^ & roundness > 0.5), more ramified resting microglia predominate in the sham, TBI control and TBI xenon groups (Fig. 6a(i)). In the right S1BF smaller round (resting) microglia predominate in the sham group and TBI control group while in the xenon group there is an increase in number of larger less round and less ramified (active) microglia (Fig. 6a(ii)). The distribution of microglia classified as resting (low activity) or active based on their size and morphology is shown in Fig. 6b. In subcortical areas where xenon attenuated neuronal loss, we observed significant (p < 0.05) increases in smaller more round low activity microglia in xenon-treated group compared to sham in left hippocampal CA1 (Fig. 6b(i)), left hippocampal CA3 (Fig. 6b(ii)), and left DG (Fig. 6b(iii)), with little or no change in the number of larger, less round and amorphous active microglia. In the right somatosensory cortex (S1BF), the median number of resting microglia in xenon-treated group was greater than sham, but this did not reach significance. In all subcortical areas except the right somatosensory cortex, the median number of low activity resting microglia was greater in the TBI xenon group compared to the TBI control group, but this did not reach significance. However in this ROI there was a significant (p < 0.05) increase in the number of active microglia in the xenon group compared to sham (Fig. 6b(iv)). In the left hypothalamus (Fig. 6b(v)), there were significant (p < 0.05) increases in both resting and active microglia in the xenon-treated group compared to the sham group.

### Xenon treatment enhances early astrocyte activation in cortical and subcortical regions

**Fig. 7:**
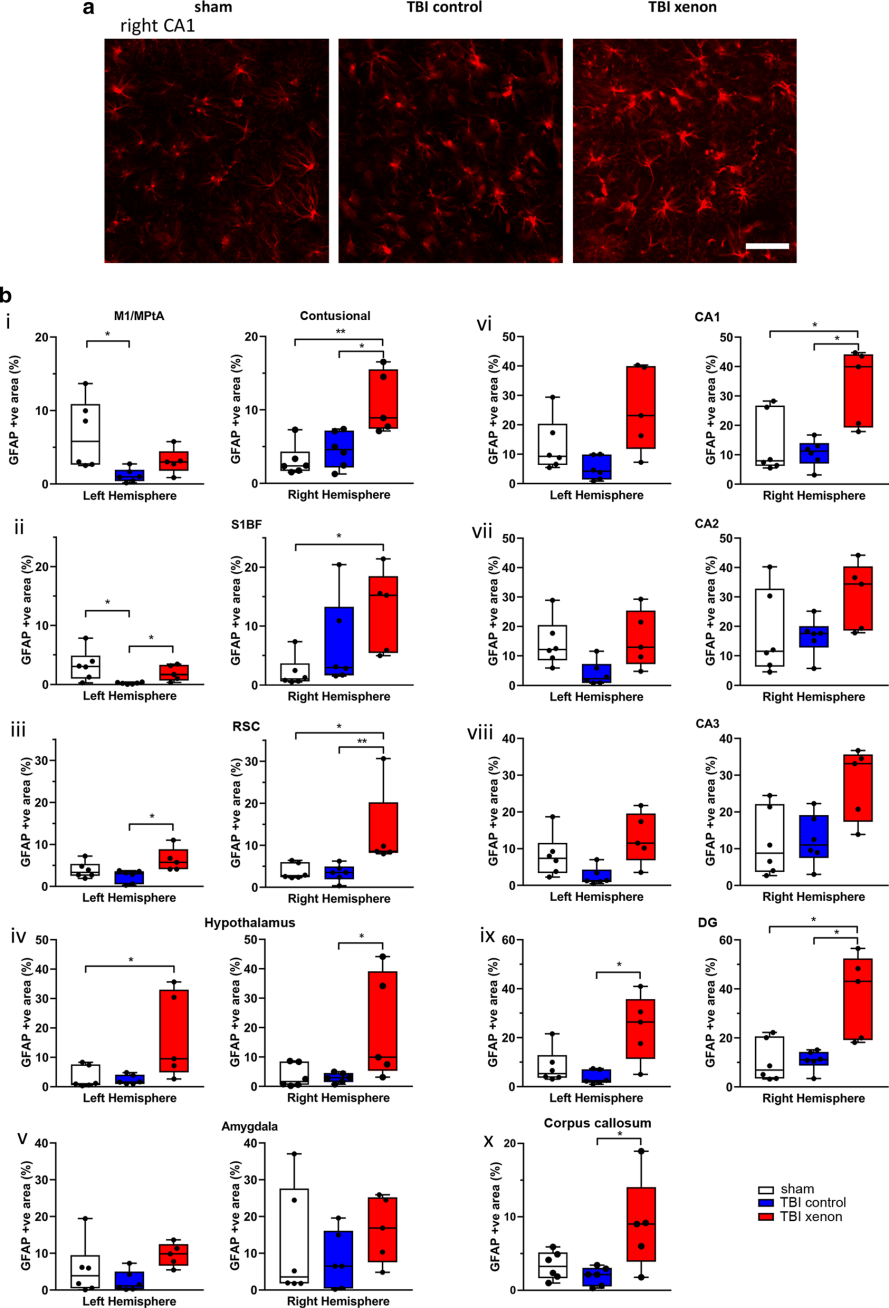
Xenon treatment enhances early astrocyte activation. **a** Typical immunostaining showing GFAP (red) staining from sham, TBI control and TBI xenon animals in right hippocampal CA1 region. The scale bar is 50 μm and applies to all images. **b** Quantification of GFAP-positive area in sham (white bars), TBI control (blue bars) and TBI xenon (red bars) in **i** motor/medial parietal association cortex (M1/MPtA) & contusional cortex, **ii** somatosensory cortex (S1BF), **iii** retrosplenial cortex (RSC) **iv** hypothalamus, **v** amygdala, **vi** hippocampal CA1, **vii** CA2, **viii** CA3, **ix** dentate gyrus (DG) and **x** corpus callosum. The lines are medians, boxes represent interquartile interval and whiskers are range. n = 6 sham (white boxes); n = 6, TBI control (blue boxes); n = 5 TBI xenon (red boxes) * p < 0.05, ** p < 0.01, compared to sham group or control TBI group as indicated by brackets, Kruskal Wallis test with Benjamini Yekutieli correction

In both the cortical and subcortical areas (Fig. 7) the GFAP-positive area was increased in the xenon-treated group. Figure 7a shows representative GFAP-positive astrocytes in the right hippocampal CA1 region from sham, TBI control and TBI xenon groups. There was a significant (p < 0.05) increase in the area of GFAP-positive astrocytes in the xenon-treated group compared to the sham group in the contusional cortex (Fig. 7b(i)), bilaterally in the somatosensory cortex (Fig. 7b(ii)), the right retrosplenial cortex (Fig. 7b(iii)), the right hippocampal CA1 and dentate gyrus (DG) subregions (Fig. 7b(vii) & (ix)). In other hippocampal subregions, the hypothalamus and amygdala bilaterally, and in the corpus callosum (Fig. 7b(iv), (v), (vii), (x)) the median GFAP-positive area in the xenon TBI group was increased but this did not reach significance compared to sham. Interestingly the GFAP positive area in the TBI xenon group was significantly increased compared to the TBI control group in the right contusional cortex, left somatosensory cortex, left and right retrosplenial cortex, right hypothalamus, right CA1, left and right dentate gyrus, and the corpus callosum (Fig. 7b(i) (ii), (iii), (iv), (vi), (ix), (x)).

## Discussion

TBI is recognized as a dynamic process starting with a mechanical force causing the primary injury and activating a complex set of pathological processes resulting in an evolving secondary injury [26]. Many of the long-term impairments in locomotor function and cognition that affect TBI survivors result from the potentially preventable secondary injury [26, 40]. Acute care of TBI patients is focused on preventing or minimizing the secondary injury that commonly develops significantly in the first 24 to 48 h after injury. Current treatment strategies are largely supportive, and clinically proven neuroprotective treatments are lacking. We have previously demonstrated xenon neuroprotection in mice after moderate TBI, [25, 41] but xenon’s efficacy in TBI had not been evaluated in a second species or other injury severities. Our aim was to evaluate the efficacy of xenon treatment following severe TBI in rats, with the focus on acute outcomes 24 h after trauma.

### Experimental model

The CCI model is a well-characterized preclinical rodent model of contusional TBI, one of the most common types of TBI in humans. The use of animal models is essential in the later stages of preclinical translation, once screening using in vitro models is complete [21, 22]. We chose a rat model because thus far xenon has been shown to be efficacious as a treatment for TBI only in mice subjected to a moderate injury [25, 41]. For the current study, we used a more severe injury [42] and investigated effects on motor function and specific cell types at an early time-point representative of the time of maximal secondary injury development.

### Xenon treatment and reduction of lesion size

We evaluated treatment with 50% xenon because this concentration would allow supplementary oxygen to be given if required, as is often the case in TBI patients. Treatment was started 30 min after injury and given for a relatively short duration of 3 h in spontaneously breathing animals, modelling a scenario where treatment could be given by first responders and continued in hospital in the neuro-ITU or during neurosurgery. Xenon treatment reduced secondary injury volume by 34%, consistent with the reduction of 38% observed in a mouse model of TBI treated with the higher concentration of 75% xenon [41].

### Xenon-treatment reduces locomotor impairment

We observed a significant reduction in locomotor speed in the TBI control group at 24 h following injury, consistent with a severe CCI injury located over the motor cortex. The reduction in speed was associated with decreased stride length; interestingly the decrease in stride length was present in all limbs and was not lateralized as might be expected from an injury on the right motor cortex. This is consistent with the neuronal loss observed in the left motor cortex that may have resulted from a contrecoup injury. However it is also possible that lack of lateralization could also be due to the ipsilateral limb compensating for the impairment in the contralateral limb. What was remarkable is that xenon-treatment following TBI attenuated both the reduction in overall speed and the reduction in stride length in all limbs. The choice of 24 h endpoint was determined by our aim of understanding the effects of xenon on neuronal loss and astroglial proliferation at this time point. Although we cannot be certain that the improvement in functional deficits at 24 h would persist at later times, our previous study in mice demonstrated that early improvement in sensorimotor function was associated with improvements in locomotor speed at 4 weeks after injury and in cognitive function 18 months after trauma [25, 41]. Although the 24 h time point is an early one for functional outcomes, our findings are nevertheless of clinical relevance because persistent reduction in walking speed and shorter stride length is observed in TBI patients [43]. Our finding that xenon treatment results in improvement in clinically relevant locomotor outcomes in rats is noteworthy.

### Xenon neuroprotection in functionally important cortical areas

We investigated neuronal loss in both pericontusional areas and brain regions in the ipsilateral and contralateral hemispheres distant from the lesion. Given our observation of improved locomotor function with xenon treatment we focused first on motor and sensorimotor areas. The primary injury was in the right motor cortex, and at 24 h this area is badly damaged, making accurate neuronal quantification impossible. In the contralateral motor cortex we observed a significant reduction neuronal cell density in the TBI control group that was prevented in the xenon-treated group. A similar neuroprotective effect of xenon across cortical layers was observed in the pericontusional right somatosensory cortex and right retrosplenial cortex, as well as the corresponding contralateral regions. Preservation of somatosensory neurons in the xenon-treated groups may also play a role in the observed improved locomotor function in this group as there is evidence that somatosensory S1 neurons can initiate motor function independent of M1 [44]. Xenon treatment resulted in neuronal preservation in pericontusional areas such as ipsilateral retrosplenial cortex and ipsilateral somatosensory cortex consistent with the reduction in lesion volume. Interestingly xenon was also effective in preserving neurons in the contralateral hemisphere that are distant from the site of impact. These findings are consistent with xenon attenuating secondary injury development and the coup contrecoup injury that is very common in human TBI. The median values of neuronal cell density in the control TBI group were significantly decreased compared to sham in many brain areas while there was no significant neuronal loss in the xenon treated group in most brain areas. An important caveat is that while the median neuronal cell density in the xenon TBI group were very similar to the sham group and were greater than in the control TBI group, the difference between the TBI groups did not reach significance except in layer 5 of the motor/association cortex. The reason for this is likely explained by the relatively small group sizes, given the variance resulting from the severe injury. Although, our observations of improvement in locomotor impairment and neuroprotection with xenon treatment in rats reported in this study are at an early time point, long-term locomotor impairment together with neuronal loss are observed in mouse moderate–severe TBI studies [45], and these can be prevented with early xenon treatment [25, 41].

### Xenon neuroprotection in specific subcortical regions

Clinical TBI is associated with impairments in cognitive function, increased anxiety and sleep disturbances; normal functioning of these behaviours is associated with subcortical brain regions. The hippocampal formation, that plays a key role in learning and memory, is known from both clinical and laboratory studies to be very sensitive to injury [46, 47]. Consistent with this, following TBI we observed bilateral hippocampal neuronal loss that was most pronounced in the ipsilateral (right) hemisphere. Interestingly, while xenon treatment was able to prevent neuronal loss in the contralateral hippocampus, in the ipsilateral hippocampus xenon treatment did not preserve neurons. The reason for this is likely due to the fact that the ipsilateral hippocampus is directly under the cortical impact site and the injury is likely to be more severe due to mechanical distortion and shear forces. In addition, this region is in direct contact with the necrotic tissue of the primary injury and will have greater exposure, both temporally and in concentration, to released amino acids and other damage associated molecular patterns (DAMPs). Neuronal loss in this region may therefore be unavoidable. Nevertheless the preservation of neurons in the contralateral hippocampus following xenon treatment is consistent with our previous observation in a mouse model of TBI [41]. Median neuronal density in the hypothalamus was reduced in the TBI control group compared to the sham group while median neuronal density in the TBI xenon group was similar to the sham. The hypothalamus is involved in regulation of sleep and pituitary function. Sleep disturbances, endocrine, and pituitary dysfunction are observed in clinical TBI and in animal models [48, 49]. Our observations of reduced hypothalamic neuronal loss with xenon treatment indicate that the effect of xenon treatment on hypothalamic function following TBI merits future investigation.

### Effect of xenon on microglia and astrocytes

The preservation of neurons in clinically relevant brain regions in the xenon-treated group was associated with an early increase in number of Iba1-positive microglia. The regions where xenon treatment resulted in pronounced neuronal preservation such as S1BF, RSC, CA1 and DG were also associated with significant increases in the number of microglia and the GFAP-positive activated astrocytes. The microglia-mediated inflammatory response can have both beneficial and detrimental aspects depending on the microglial activation state. There is accumulating molecular evidence that microglial activation state is more complex than a simple binary model [50]. Nevertheless in TBI, while it appears that chronic long term microglial activation is harmful, there is evidence that at early time-points microglia are beneficial and assist in clearing debris [50]. Our findings suggest that xenon is preferentially promoting a proliferation of small round low activity or resting microglia, rather than larger amorphous less ramified microglia characteristic of the harmful M1 activated phenotype. In some regions such as the right S1BF and left hypothalamus we observed an increase in both active and resting microglia in the xenon-treated group. The right S1BF is a pericontusional region and the increase in active microglia may represent activation due to proximity to necrotic tissue in the contusion or may represent migration of active microglia toward the contusion. An increase in overall number and number of active microglia following TBI has been observed in previous studies [36, 37] and xenon appears to enhance this homeostatic response. It is of note that in the right S1BF and bilaterally in the hypothalamus there is also increased astrogliosis in the xenon-treated group, as there is recent evidence that activated microglia are able to promote neuroprotective reactive astrocytes [51, 52]. Our findings at 24 h after injury of neuronal preservation associated with an increase in number of microglia and astrocyte activation are consistent with microglia promoting repair and regeneration mediated by neuroprotective reactive astrocytes. Given the dual nature of the inflammatory response, it has been suggested that therapeutic interventions should promote the early helpful inflammation, while preventing the chronic neuroinflammation associated with late-onset cognitive impairment and dementia [50]. Our current findings in rats, together with our recent observation that xenon treatment prevented chronic neuroinflammation, and long-term cognitive impairment 18 months after TBI in mice [41], suggest that xenon may have such a profile.

## Conclusions

Our aim was to evaluate the potential of xenon as a neuroprotectant for treatment of TBI in a rat model of severe TBI. We have previously shown that xenon is effective in a mouse model of moderate TBI [25, 41], but before clinical translation it is of utmost relevance and usually a requirement to demonstrate efficacy in a second species. As clinical TBI severity is heterogeneous, it is also important to evaluate neuroprotection in different injury severities [26]. In addition to assessing clinically relevant locomotor outcomes we aimed to do a more complete characterization and determine cellular effects of xenon treatment in brain regions associated with a variety of functional impairments that are common following TBI. Our study was carried out following the ARRIVE guidelines [27] and an important aspect was the randomization of animals to treatment group and that all functional and histological measurements were made by blinded observers. Xenon prevented or reduced neuronal loss in motor/association cortex and sensorimotor cortex, associated with locomotor and sensory deficits, and in the hippocampus and retrosplenial cortex, associated with cognitive impairments. We used a translationally relevant concentration of xenon that would allow supplementary oxygen to be given if required. Treatment start time was 30 min after injury, with a relatively short duration of 3 h, modelling a scenario where xenon-treatment could be initiated by first responders and continued in the early hospital phase. We observed significant neuroprotective effects on functional and cellular outcomes with only 3 h treatment duration, and it is plausible that further improvement could be observed with longer treatment duration, given our previous striking findings showing very long term benefit in mice [41]. In our current study xenon treatment was given at normothermia. Xenon has been reported to act synergistically with cooling in models of ischemic brain injury [13] and it is possible that xenon’s efficacy in TBI may be enhanced by combining it with mild to moderate hypothermia. There are currently no clinically proven treatments specifically targeting acute neuronal loss after TBI [53]. Xenon is approved for clinical use as a general anesthetic and has recently completed clinical trials for ischemic brain injury after neonatal hypoxic-ischemic encephalopathy and cardiac arrest in adults.[20, 54–56]. Our current findings demonstrate for the first time in rats that xenon improves functional outcome and prevents neuronal loss. This study, together with our previous studies in mice, support the view that xenon could be an early neuroprotective treatment for TBI.

## Data Availability

The datasets used and/or analyzed during the current study are available from the corresponding author on reasonable request.

## Abbreviations

CCI: Controlled cortical impact
DAPI: 4′,6-Diamidino-2-phenylindole
GFAP: Glial fibrillary acidic protein
M1: Primary motor cortex
Iba1: Ionized calcium binding adaptor molecule
MRI: Magnetic resonance imaging
MPtA: Medial parietal temporal area
NeuN: Neuronal nuclear protein
NMDA: N-methyl-d-aspartate
PBS: Phosphate buffered saline
RSC: Retrosplenial cortex
ROI: Region of interest
S1BF: Primary somatosensory cortex barrel field
SEM: Standard error of mean
TBI: Traumatic brain injury
